# European *Aedes albopictus* and *Culex pipiens* Are Competent Vectors for Japanese Encephalitis Virus

**DOI:** 10.1371/journal.pntd.0005294

**Published:** 2017-01-13

**Authors:** Mélissanne de Wispelaere, Philippe Desprès, Valérie Choumet

**Affiliations:** 1 Unité Environnement et Risques Infectieux, Département Infection et Epidémiologie, Institut Pasteur, Paris, France; 2 Université de La Réunion, INSERM U1187, CNRS UMR 9192, IRD UMR 249, Unité mixte 134 Processus Infectieux Insulaire Tropical (PIMIT), plateforme technologique CYROI, Sainte-Clotilde, La Réunion, France; INDEPENDENT RESEARCHER, UNITED STATES

## Abstract

**Background:**

Japanese encephalitis virus (JEV) is the causative agent of Japanese encephalitis, the leading cause of viral encephalitis in Asia. JEV transmission cycle involves mosquitoes and vertebrate hosts. The detection of JEV RNA in a pool of *Culex pipiens* caught in 2010 in Italy raised the concern of a putative emergence of the virus in Europe. We aimed to study the vector competence of European mosquito populations, such as *Cx*. *pipiens* and *Aedes albopictus* for JEV genotypes 3 and 5.

**Findings:**

After oral feeding on an infectious blood meal, mosquitoes were dissected at various times post-virus exposure. We found that the peak for JEV infection and transmission was between 11 and 13 days post-virus exposure. We observed a faster dissemination of both JEV genotypes in *Ae*. *albopictus* mosquitoes, when compared with *Cx*. *pipiens* mosquitoes. We also dissected salivary glands and collected saliva from infected mosquitoes and showed that *Ae*. *albopictus* mosquitoes transmitted JEV earlier than *Cx*. *pipiens*. The virus collected from *Ae*. *albopictus* and *Cx*. *pipiens* saliva was competent at causing pathogenesis in a mouse model for JEV infection. Using this model, we found that mosquito saliva or salivary glands did not enhance the severity of the disease.

**Conclusions:**

In this study, we demonstrated that European populations of *Ae*. *albopictus* and *Cx*. *pipiens* were efficient vectors for JEV transmission. Susceptible vertebrate species that develop high viremia are an obligatory part of the JEV transmission cycle. This study highlights the need to investigate the susceptibility of potential JEV reservoir hosts in Europe, notably amongst swine populations and local water birds.

## Author Summary

Japanese encephalitis virus (JEV) is the leading cause of viral encephalitis in Asia. JEV is maintained in a cycle involving mosquitoes and vertebrate hosts, mainly pigs and wading birds. Humans can be infected when bitten by an infected mosquito. *Culex tritaeniorhynchus* is the main vector of the disease in tropical and subtropical areas. The recent detection of JEV in birds and mosquitoes collected in Northern Italy has led us to evaluate the putative emergence of this arboviral disease in Europe. For this purpose, we have tested the competence of European populations of *Cx*. *pipiens* and *Aedes albopictus* to transmit this virus in a laboratory setting. We showed that these local mosquitoes could be infected and were capable of transmitting a pathogenic virus to mice. It is thus urgent to evaluate the risks of JEV emergence in European regions displaying a favorable environment for mosquito vectors, susceptible pigs and wading birds.

## Introduction

Japanese encephalitis is one of the major viral encephalitides in Asia, with an estimated 68,000 human cases per year [1]. Up to 30% of the symptomatic cases are fatal, and long-term neurologic *sequelae* can occur in 30 to 50% of survivors [2]. Japanese encephalitis virus (JEV) is the causative agent of Japanese encephalitis, and is transmitted through the bite of an infected mosquito. JEV is a member of the *Flavivirus* genus in the *Flaviviridae* family and has a positive-sense RNA genome. The viral polyprotein is processed into 10 proteins: three structural proteins and seven nonstructural proteins. JEV strains can be differentiated into five genotypes (1 to 5) based on phylogenetic studies of the viral envelope protein sequences. Until recently, most of the strains of JEV at the origin of major epidemics in the South, East and Southeast Asia regions belonged to genotype 3 [3, 4]. Recently a shift in prevalence from JEV genotype 3 to 1 has been observed in several Asian countries [5–7]. JEV genotype 5 was first isolated in Malaysia in 1952, and is genetically and serologically distinct from other genotypes [8–10]. No other JEV genotype 5 strain had been identified until its recent isolation from *Culex* spp. mosquito pools in China in 2009 [11] and in South Korea in 2010 and 2012 [12, 13].

Most of the vectors for JEV belong to the *Culicinae* subfamily in the *Culicidae* family. In most Asian countries, the main vector is *Culex tritaeniorhynchus* [7, 14–18], while *Cx*. *annulirostris* was identified as the main vector for JEV transmission in Australia [19, 20]. Several secondary vectors are known to efficiently transmit JEV: *Cx*. *annulirostris*, *Cx*. *annulus*, *Cx*. *fuscocephala*, *Cx*. *gelidus*, *Cx*. *sitiens* or *Cx*. *vishnui*. The fact that JEV can be detected in field-caught mosquitoes belonging to numerous species, such as *Cx*. *pipiens* [12, 17, 21], *Aedes albopictus* [7, 22], or *Anopheles* species [7, 23], poses the question if those mosquito species could also act as secondary vectors for JEV.

The JEV enzootic cycle involves mosquitoes and amplifying vertebrate hosts, such as water birds and domestic swine [24]. Humans are considered as dead-end hosts, while they can be infected by JEV, they do not develop high levels of blood viremia, and thus cannot infect mosquitoes [25].

A fragment of JEV genome was detected in a pool of *Cx*. *pipiens* and in birds caught in 2000 and 2010 in Northern Italy [21, 26] raising the threat of a putative emergence of the virus in Europe [27]. Recent studies have shown that *Ae*. *detritus* from England and *Ae*. *japonicus japonicus* from Germany were competent to transmit JEV [28, 29]. These observations emphasize on the need to study the vector competence of European mosquito populations for JEV. *Ae*. *albopictus* is currently expanding its range, predominantly in temperate areas in North America and Europe, and this invasion raises a public health threat for pathogens transmitted by this vector, such as Zika and dengue viruses. *Cx*. *pipiens* is the most widely distributed species of mosquito in the world, and is typically found in temperate regions. *Cx*. *pipiens* complex mosquitoes play important roles in the transmission of several medically relevant pathogens such as West Nile virus (WNV), Saint Louis encephalitis virus, and filarial worms [30–32].

In the present study, we evaluated the competence of *Ae*. *albopictus* and *Cx*. *pipiens* populations collected in the South of France for two representative strains of JEV, belonging to distinct genotypes. We found that both viruses could infect and disseminate to high efficiency in either vector and could be readily transmitted. We additionally evaluated the influence of mosquito salivary factors on viral pathogenesis and showed that they had no impact on the development of Japanese encephalitis in a mouse model for the disease. Overall, these findings highlight the need for investigation of the other factors that could contribute to JEV emergence in Europe.

## Methods

### Ethics statement

The protocols and subsequent experiments were ethically approved by the Ethic Committee for Control of Experiments on Animals (CETEA) at the Institut Pasteur and declared to the French Ministère de l’Enseignement Supérieur et de la Recherche (n° 000762.1) in accordance with European regulations. Experiments were conducted following the guidelines of the Office Laboratory of Animal Care at the Institut Pasteur. Euthanasia was performed by CO2 asphyxiation, followed by cervical dislocation. Anesthesia was performed by intraperitoneal injection of a mixture of Xylazine (Rompun, 5 to 10 mg/kg) and Kétamine (Imalgène, 80 to 100 mg/kg).

### Mosquito rearing

*Cx*. *pipiens* form *pipiens* and *Ae*. *albopictus* mosquito colonies were established in the laboratory using mosquitoes collected in Montpellier and Nice, in 2010 and 2011, respectively. Eggs of each mosquito colony were hatched in tap water. *Larvae* were reared in plastic trays containing tap water supplemented with brewer’s yeast tablets and cat food. Adults were maintained at 27°C, 80% relative humidity with a 12 h:12 h light: dark cycle and were given continuous access to 10% sucrose solution.

### Cells

Mosquito *Ae*. *albopictus* C6/36 cells were maintained at 28°C in Leibovitz medium (L15) supplemented with 10% heat-inactivated fetal bovine serum (FBS). Baby hamster kidney-derived BHK-21 (purchased from ATCC), chicken fibroblast-derived DF-1 (obtained from Nadia Naffakh), and human kidney-derived HEK293T cells (purchased from ATCC) were maintained at 37°C in DMEM supplemented with 10% FBS.

### Antibodies

Mouse hybridomas producing the monoclonal antibody 4G2 anti-Flavivirus E were purchased from ATCC and a highly purified antibody preparation was produced by RD Biotech. The anti-mosquito saliva antibody was produced in house in rabbits exposed to mosquito bites. Horseradish peroxidase (HRP)-conjugated goat anti-mouse and anti-rabbit IgG antibodies were obtained from Bio-Rad Laboratories. Alexa Fluor 488-conjugated goat anti-mouse IgG antibody was obtained from Jackson ImmunoResearch.

### Production of recombinant JEV

A molecular cDNA clone of JEV genotype 3 strain RP-9 was kindly provided by Yi-Lin Ling and was modified as described previously [33]. A molecular cDNA clone of JEV genotype 5 strain XZ0934 was described previously [33].

To produce infectious virus, the molecular clones were transfected into HEK293T cells using Lipofectamine 2000 (ThermoFischer Scientific). At 3 days post-transfection, viral supernatants were collected and used to infect DF-1 cells in order to grow final virus stocks for experiments.

### Virus infections

For infections, C6/36 cells were seeded in 24-well tissue culture plates in L15, supplemented with 2% FBS. Aliquots of virus were diluted in 200 μl of medium and added to the cells. Plates were incubated for 1 h at 28°C. Unadsorbed virus was removed by two washes with Dulbecco's phosphate-buffered saline (DPBS) and then 1 ml of L15 supplemented with 2% FBS was added to the cells, followed by incubation at 28°C until collection.

### Focus forming assay (FFA)

BHK-21 cells were seeded in 24-well plates. Tenfold dilutions of virus samples were prepared in duplicate in DMEM and 200 μl of each dilution was added to the cells. The plates were incubated for 1 h at 37°C. Unadsorbed virus was removed, after which 1 ml of DMEM supplemented with antibiotics and antifungals, 1.6% carboxymethyl cellulose (CMC), 10 mM HEPES buffer, 72 mM sodium bicarbonate, and 2% FBS was added to each well, followed by incubation at 37°C for 32 h. The CMC overlay was aspirated, and the cells were washed with PBS and fixed with 4% paraformaldehyde for 15 min, followed by permeabilization with 0.1% Triton-X100 for 5 min. After fixation, the cells were washed with PBS and incubated for 1 h at room temperature with anti-E antibody (4G2), followed by incubation with HRP-conjugated anti-mouse IgG antibody. The assays were developed with the Vector VIP peroxidase substrate kit (Vector Laboratories) according to the manufacturer’s instructions. The viral titers were expressed as focus forming units (FFU)/ml.

### Oral infection of mosquitoes and dissections

Seven day-old female mosquitoes were deprived of sucrose 24 h prior to the infectious blood meal. They were then allowed to feed for 2 h on blood-soaked cotton pledgets in the dark at 28°C. The infectious blood meal was comprised of washed rabbit erythrocytes (obtained from animals housed at the Institut Pasteur animal facility), viral suspension, and ATP (as a phagostimulant) at a final concentration of 5 μM. The virus titer in the blood meal was adjusted to 8 x 10^6^ FFU/ml. Blood-fed females were sorted and transferred into cardboard containers covered with mosquito nets. After exposure, engorged mosquitoes were maintained at 26°C, 80% relative humidity, with a 10 h: 10 h light: dark cycle with simulation of dawn and sunset during 2 h. Mosquitoes were dissected at various time points after oral exposure. For titrations, the mosquitoes or individual organs were collected in a tube containing 0.5 mm glass beads and 300 μl of DMEM supplemented with 2% FBS. The organs were ground for 30 sec at maximum speed, using a Minilystissue homogeneizer (Bertin) and stored at -80°C until analysis. Experiments were reproduced twice with 5 to 10 mosquitoes collected at each time point for dissection.

### Mosquito salivation

JEV exposed mosquitoes were anesthetized at 4°C, legs and wings were removed and the bodies were attached to a glass slide using double-sided tape. The proboscis was manually inserted into a 10 μl low binding pipette tip filled with 10 μl DMEM containing 2% FBS. The tip contents were collected 30 min later in a tube. Two μl were transferred to a tube containing 2 μl SDS sample buffer and analyzed by dot-blot to verify the presence of saliva. Four μl were analyzed by FFA to determine virus titer. Ten to 20 mosquitoes were analyzed for each time point (days 11, 12 and 13 post-virus exposure) and experiments were reproduced twice.

### Salivary gland extracts preparation

Five days after emerging, mosquito females were blood-fed on mice previously anesthetized by intraperitoneal injection of a mixture of Xylazine (5 to 10 mg/kg) and Ketamine (80 to 100 mg/kg). Three weeks later, 100 salivary glands (SG) were dissected and placed in 100 μl 1X PBS. SG extracts were prepared by sonicating the SG (five times at 4 min each with a pulse ratio of 2 sec on / 2 sec off) and centrifuging the crude extract at 10,000 g for 15 min at 4°C. The supernatant was transferred to clean tubes and stored at −80°C. The inocula used in our experiments contained the equivalent to a pair of SG.

### Western blotting

Protein lysates were prepared by cell lysis in radio-immunoprecipitation assay (RIPA) buffer (Bio Basic) containing protease inhibitors (Roche). Equal amounts of proteins were loaded on a NuPAGE Novex 4–12% Bis-Tris protein gel (ThermoFisher Scientific) and transferred to a polyvinylidene difluoride membrane (Bio-Rad) using the Trans-Blot Turbo Transfer System (Bio-Rad). After blocking the membrane for 1 h at room temperature in PBS-Tween (PBS-T) plus 5% milk, the blot was incubated overnight at 4°C with appropriate dilutions of the primary antibodies. The membrane was then washed in PBS-T and then incubated for 1 h at room temperature in the presence of HRP-conjugated secondary antibodies. After washes in PBS-T, the membrane was developed using Pierce ECL Western Blotting Substrate (ThermoFisher Scientific) and exposed to film.

### Dot-blot on mosquito saliva

The saliva collected from each mosquito was blotted onto a nitrocellulose membrane. Two μl of DMEM containing 2% FBS and 1 μg of mosquito salivary gland extract were deposited on the membrane as negative and positive controls, respectively. The membranes were blocked for 1 h in PBS-T plus 5% milk and incubated overnight at 4°C with an anti-mosquito saliva antibody. The blots were then processed as indicated above for Western blotting.

### Immunofluorescence analysis (IFA)

After dissection, midguts (MG) and salivary glands (SG) were placed on slides and the PBS removed. MG were fixed in acetone for 15 min. SG were fixed in 4% paraformaldehyde for 15 min. Both slides were dried and stored at 4°C until use. The MG and SG were then rehydrated in PBS for 15 min. The MG and the SG were incubated in Triton X100 (0.2%) for 2 h and 15 min, respectively. They were then washed with PBS and incubated for 30 min with PBS + 0.1% Tween 20 containing 1% BSA. The slides were drained and incubated overnight at 4°C with anti-flavivirus protein E 4G2 antibody diluted in PBS, then washed with PBS. The slides were next incubated for 1 h with a fluorophore conjugated antibody, and washed with PBS. After washing, a drop of ProLong Gold Antifade reagent with DAPI (ThermoFisher Scientific) was placed on each slide and a cover slide was added. All preparations were examined using a fluorescence microscope (Axioplan 2 Imaging, Zeiss).

### Mice experiments

Three-week-old female BALB/c mice were housed under pathogen-free conditions at the Institut Pasteur animal facility. Groups of mice were anesthetized as described above, and were next intradermally inoculated with 50 FFU of JEV genotype 5 in absence or in presence of salivary gland extract or with JEV-infected saliva diluted in 100 μl of DPBS supplemented with 0.2% endotoxin-free serum albumin.

### Statistical analysis

An unpaired t test was used to compare quantitative data, and a Log-rank (Mantel-Cox) test was used to compare survival data. GraphPad Prism was used for all statistical analysis.

### Animal handling

Rabbit and mice were housed in Institut Pasteur animal facilities.

## Results

### Vector competence of European strains of mosquitoes for JEV

**Fig 1:**
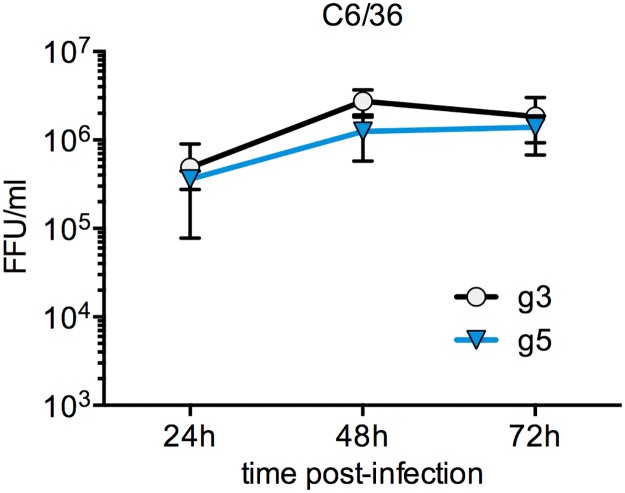
Kinetics of JEV g3 and g5 infection *in vitro*. *Ae*. *albopictus* derived C6/36 cells were infected with JEV g3 or g5 at a MOI of 1. The infectious virus released to the supernatants at 24, 48 and 72 h post-infection was quantified by FFA in BHK-21 cells. The error bars represent the standard deviation between two independent experiments (in each experiment, titrations were done on duplicate experimental samples). FFU, focus forming units.

To assess the vector competence of European mosquitoes for JEV, we decided to use two molecular clones of viruses (RP-9 and XZ0934), which are representative of two currently circulating genotypes. The well-characterized genotype 3 strain, JEV RP-9, was isolated from *Cx*. *tritaeniorhynchus* mosquitoes in Taiwan in 1985 [34, 35], while the genotype 5 strain, JEV-XZ0934, was recently isolated from *Cx*. *tritaeniorhynchus* mosquitoes in China in 2009 [11]. For simplification, JEV-RP-9 and JEV-XZ0934 will be hereafter referred to as JEV g3 and JEV g5, respectively. Both viruses were produced by transfection of cDNA into mammalian cells, as previously described [33], followed by amplification of viral stocks in chicken fibroblasts DF-1 cells. Those viruses displayed comparable growth after infection of *Ae*. *albopictus* derived C6/36 cells (Fig 1, [33]).

To evaluate the vector competence of European mosquito species for JEV, we exposed mosquitoes to either JEV g3 or JEV g5 by feeding on blood meals containing approximately 8 x 10^6^ FFU of virus per ml. We note that the viremia in infected pigs or in birds can reach up to 10^7^ PFU/ml, but is on average 10^4^ PFU/ml [36–41]. While we offered blood meals that contained relatively high levels of virus, it is generally accepted that a greater quantity of virus is needed to infect mosquitoes orally with artificial mixtures than with viremic hosts [42]. For each experiment, 3 blood-fed mosquitoes were harvested immediately post-virus exposure, and the ingested virus titers were evaluated by FFA. The amount of ingested infectious virus was comprised between 400 and 9,000 FFU per mosquito, with an average titer of 4,000 FFU.

Previous studies on vector competence of various species of mosquitoes for JEV have shown the peak for JEV infection and transmission occurs between 5 and 23 days after peroral infection [23, 29, 43–48]. Our preliminary studies showed that, under our experimental conditions, the majority of *Cx*. *pipiens* and *Ae*. *albopictus* were infected from 10 to 15 days post-virus exposure. We chose to focus collection times around the peak of viral transmission and harvested samples at 7, 11, 12 and 13 days post-virus exposure. We note that the survival rate of exposed mosquitoes dropped considerably after 2 weeks of infection, and consequently did not analyze the levels of mosquito infection beyond this point.

**Fig 2:**
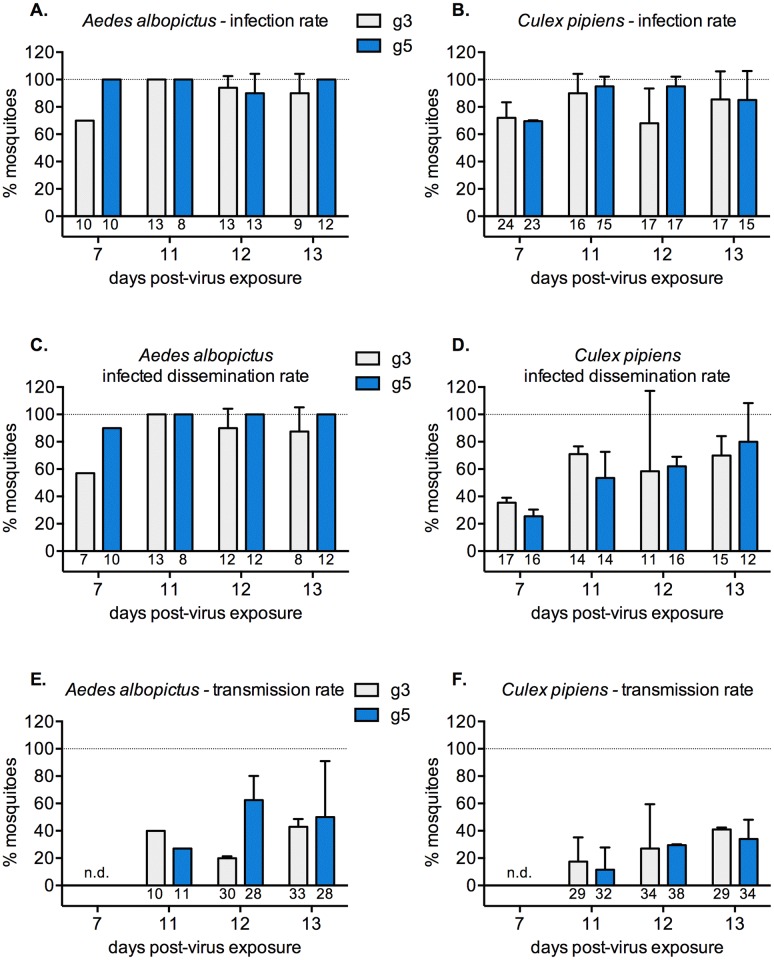
Vector competence of European strains of mosquitoes for two different JEV genotypes following feeding on infectious bloodmeals. Infection rates in *Ae*. *albopictus* (A) or *Cx*. *pipiens* (B) mosquitoes exposed to either JEV g3 or JEV g5 by feeding on blood meals containing 8 x 10^6^ FFU/ml of virus. The infection rates were determined after titration of midguts harvested from mosquitoes on days 7, 11, 12 and 13 post-virus exposure. The infected dissemination rates (C and D) were calculated by titration of the heads of infected mosquitoes. The transmission rates (E and F) were calculated by titration of the saliva collected from all blood-fed mosquitoes. The error bars represent the standard deviation between two independent experiments. The number of mosquitoes analyzed for each condition is indicated below each graph bar. An unpaired t test was employed to determine significant differences between JEV genotypes at each time point. No statistically significant differences were found (P≥0.08). n.d. not determined.

First, we determined the infection rates in *Ae*. *albopictus* (Fig 2A) and *Cx*. *pipiens* (Fig 2B) mosquitoes by titrating the midguts harvested from mosquitoes. Next, we measured the levels of JEV infection in the heads of infected mosquitoes, and calculated infected dissemination rates (Fig 2C and 2D). We did not observe any statistically significant differences in infection rates amongst genotypes for each mosquito species or by time after the infectious blood meal. We did note that dissemination of JEV was faster in *Ae*. *albopictus* mosquitoes, when compared with *Cx*. *pipiens* mosquitoes. Notably, at 7 days post-virus exposure, we found that 57 to 90% of *Ae*. *albopictus* mosquitoes were systemically infected, whereas only 26 to 36% of *Cx*. *pipiens* were (Fig 2C and 2D). Last, we determined transmission rates through titration of saliva collected from blood-fed mosquitoes (Fig 2E and 2F). While this is a method widely used to determined transmission rates, we observed that salivation assays are highly dependent on salivation efficiency, and that the levels of virus in saliva can sometimes be below the detection limit of our titration assay. Keeping in mind that this determination of the virus transmission rates has limitations, we observed that both mosquito species transmitted JEV at rates ranging from 20 to 63% for *Ae*. *albopictus*, and from 12 to 41% for *Cx*. *pipiens* (Fig 2E and 2F).

### Characterization of JEV infection in European strains of mosquitoes

**Fig 3:**
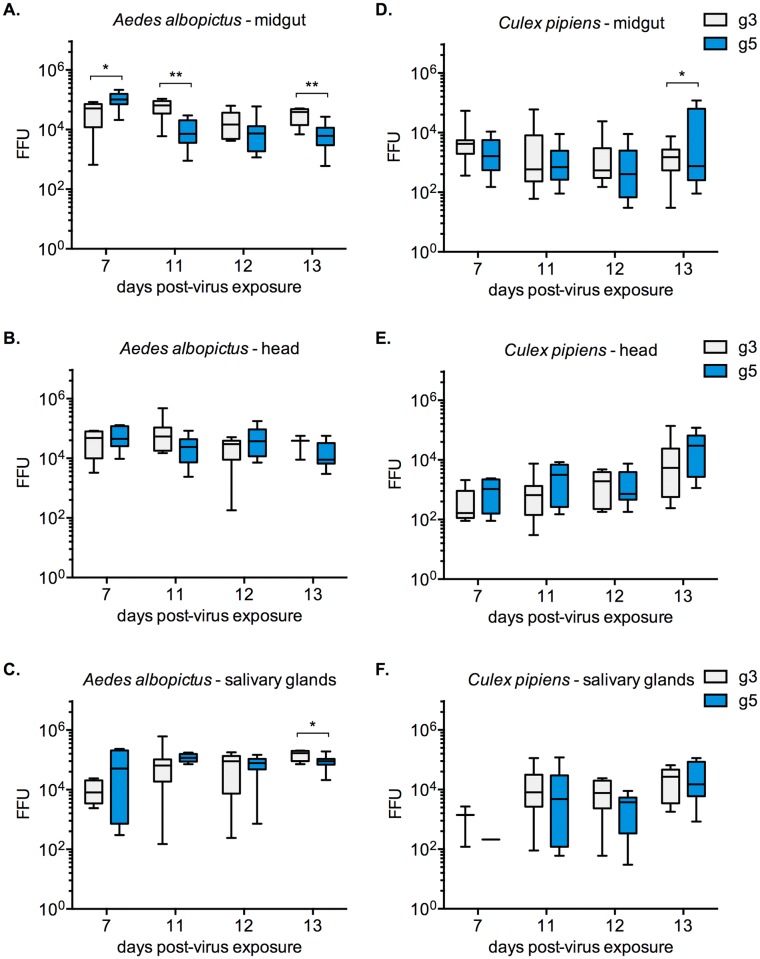
Kinetics of JEV infection in European strains of *Ae*. *albopictus* and *Cx*. *pipiens* mosquitoes. *Ae*. *albopictus* (A, B and C) or *Cx*. *pipiens* (D, E and F) mosquitoes were exposed to either JEV g3 or JEV g5 by feeding on blood meals containing 8 x 10^6^ FFU/ml of virus. At 7, 11, 12 and 13 days post-virus exposure, the midguts (A and D), heads (B and E) and salivary glands (C and F) were harvested from individual mosquitoes and the levels of infectious virus in each organ was quantified by FFA in BHK-21 cells. The error bars represent the standard deviation amongst infected samples collected from two independent experiments. An unpaired t test was employed to determine significant differences between JEV genotypes at each time point (***, P < 0.001; **, 0.001 < P < 0.01; *, 0.01 < P < 0.05; only statistically significant differences are shown).

Next, we analyzed the levels of JEV g3 and g5 accumulation in the different mosquito organs that had been harvested (Fig 3). We noted that JEV levels in the midguts slowly decreased between 7 and 13 days post-virus exposure, while viral levels in heads and salivary glands increased over time, which is consistent with patterns of viral dissemination in mosquitoes. We noted that at 7 days post-virus exposure, the rates of salivary glands infection ranged from 40 to 80% for *Ae*. *albopictus*, and from 5 to 9% for *Cx*. *pipiens*. Viral infection of salivary glands has been shown to correlate well with infection of saliva [43], and thus we hypothesize that *Ae*. *albopictus* mosquitoes were likely to transmit JEV at earlier times than *Cx pipiens*. Interestingly, in *Ae*. *albopictus* mosquitoes midguts, we observed a significant difference in the titers of JEV g5 when compared to JEV g3 titers (Fig 3A). Notably, it appeared that JEV g5 accumulated to higher levels than JEV g3 at 7 days post-virus exposure, and to lesser levels at later infection times (11 to 13 days post-virus exposure).

**Fig 4:**
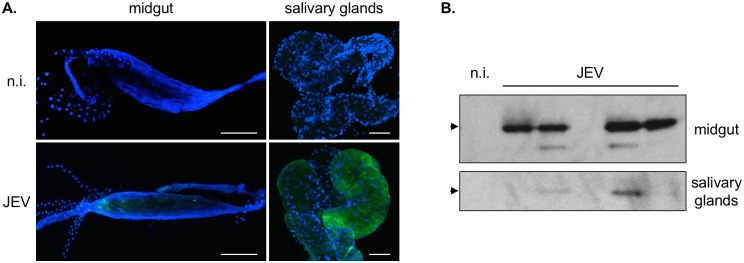
Analysis of JEV infection in mosquitoes. A. Visualization of JEV envelope protein in the midgut (left panel) and salivary glands (right panel) of *Ae*. *albopictus* mosquitoes infected with JEV g3 at 14 days post-feeding on an infectious blood meal. Samples obtained from non-infected mosquitoes were used as a control (n.i.). Scale bars: 500 μm for the midgut samples, 50 μm for the salivary glands samples. B. Detection of JEV envelope protein in lysates obtained from midguts (top) and salivary glands (bottom) harvested from mosquitoes at 11 days post-feeding on an infectious blood meal. Samples from non-infected mosquitoes are used as a control (n.i.). The expected size for JEV envelope protein is indicated with an arrow.

Additionally, we analyzed the distribution of JEV envelope protein in the organs of infected mosquitoes (Fig 4). First, we performed immuno-localization within organs harvested from *Ae*. *albopictus* mosquitoes at 14 days post-virus exposure, which corresponds to a peak in viral transmission (Fig 4A). While envelope protein staining within the midgut was relatively weak, there was a strong staining of numerous cells within both lobes of salivary glands. Samples collected at 11 days post-virus exposure were also analyzed by western blotting and showed good detection of the envelope protein in midguts and salivary glands (Fig 4B).

### Detection of JEV in infected European mosquito saliva

**Fig 5:**
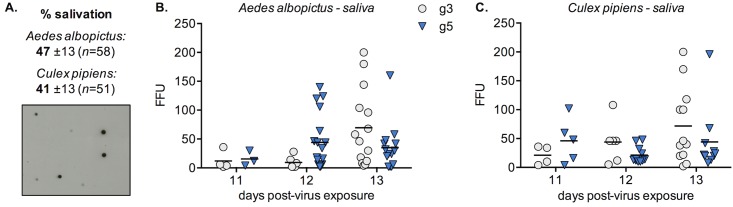
Analysis of salivas collected from European mosquitoes infected by different genotypes of JEV. At 11, 12 and 13 days post-virus exposure, salivas were collected from mosquitoes *via* forced salivation in pipette tips. A. Individual salivation samples were analyzed in a dot-blot assay using an anti-saliva antibody, to determine the salivation efficiency for each mosquito species. The rates of salivation obtained from two independent salivation assays are given, and a representative dot blot assay is shown. B. and C. The levels of infectious virus in JEV-positive saliva were measured by FFA in BHK-21 cells.

To evaluate the levels of virus secreted in mosquito saliva, we collected mosquitoes at 11, 12 and 13 days post-feeding on an infectious blood meal, and performed forced salivation. Since not all of the mosquitoes salivate when subjected to this assay, we also performed a survey of successful salivation. A fraction of the collected saliva was dotted on a membrane, and was next incubated with an antibody specific for mosquito saliva (Fig 5A). We noted that both mosquito species efficiently salivated under our experimental conditions, and that the levels of actual salivation were above 40% for either mosquitoes (Fig 5A). The collected saliva was then subjected to a standard infectivity assay to determine the levels of JEV transmitted in JEV-positive saliva at each time point (Fig 5B and 5C). We noted that for both mosquito species, higher levels of virus were secreted in saliva at later times post-virus exposure, which mirrored the increase in viral load in salivary glands (Fig 3C and 3F). The levels of infectious virus in saliva ranged between 2 and 200 FFU for JEV g3 (45 and 55 FFU in average for *Ae*. *albopictus* and *Cx*. *pipiens*, respectively), and between 2 and 196 FFU for JEV g5 (38 and 35 FFU in average for *Ae*. *albopictus* and *Cx*. *pipiens*, respectively).

### European mosquito saliva or salivary glands do not enhance JEV pathogenesis in a mouse model for JEV disease

**Fig 6:**
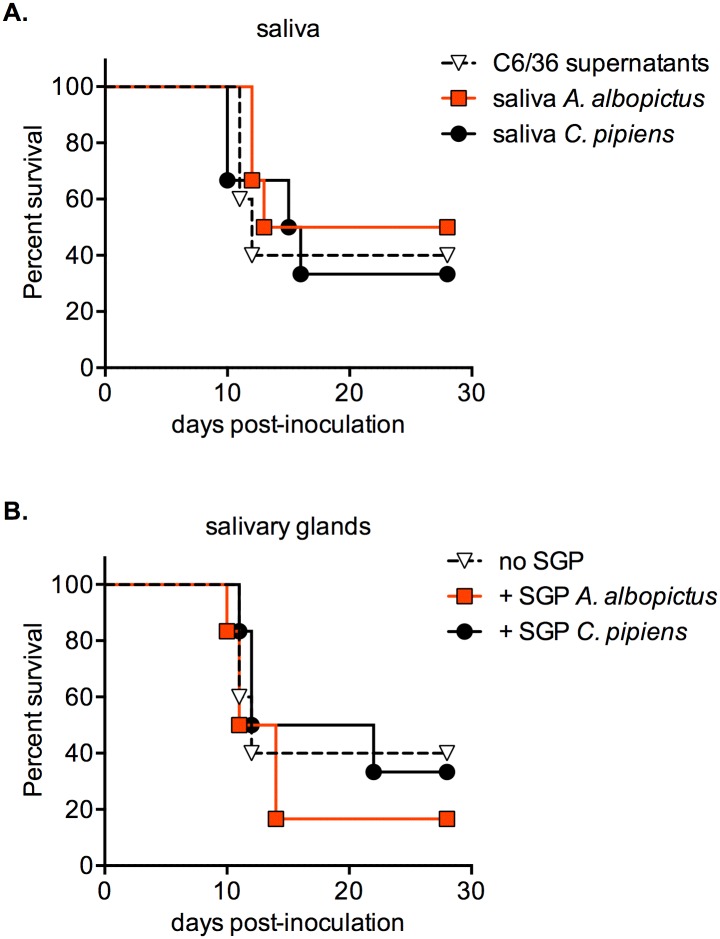
Effect of mosquito saliva and salivary glands on JEV infection in a mouse model. Groups of 3-week-old BALB/c mice were monitored for survival after intradermal injection with JEV g5 (*n* = 6 per group). A. Mice were injected with JEV-positive saliva collected from infected mosquitoes. The levels of virus in saliva were between 7 and 98 FFU (*Ae*. *albopictus*: 9, 15, 22, 28, 60 and 70 FFU; *Cx*. *pipiens*: 7, 16, 24, 30, 51 and 98 FFU). Control mice were injected with 50 FFU of virus grown in C6/36 cells (C6/36 supernatants). B. Mice were injected with 50 FFU of virus, in presence or absence of one pair of salivary glands (SGP) harvested from either *Ae*. *albopictus* or *Cx*. *pipiens* mosquitoes. A Log-rank (Mantel-Cox) test was employed to determine significant differences between *inocula*. No statistically significant differences were found (P≥0.6).

Next we assessed whether the virus transmitted by European mosquitoes was capable of developing a productive infection in mammalian hosts. To evaluate this, we used a previously characterized murine model for Japanese encephalitis, based on JEV g5 infection of 3-week-old BALB/c mice [33]. Three-week-old BALB/c mice were injected *via* intradermal route with JEV, as this mode of injection most resembles a mosquito bite. First, JEV-positive saliva samples collected from *Ae*. *albopictus* and *Cx*. *pipiens* mosquitoes (Fig 5) were used as an inocula (Fig 6A). Saliva containing various loads of virus was used, with a titer comprised between 7 and 98 FFU. A control group of mice were similarly injected with JEV grown from C6/36 cells, using a single dose of 50 FFU (Fig 6A). As expected, the animals rapidly exhibited limb paralysis and encephalitis. We did not observe any significant differences in survival rates amongst the different *inocula* (Fig 6A). The survival rate was between 33 and 40%, with a mean survival time of 11 to 12.5 days.

The detection of JEV-specific antibodies showed that all surviving mice had been exposed to the virus (S1 Fig).

Since it was shown that mosquitoes can inject salivary components that influence the outcome of viral infection [49–51], we next evaluated the impact of European mosquito salivary glands on JEV pathogenesis in a murine model. As described above, we used the intradermal route to inject 50 FFU of JEV g5 to 3-week-old BALB/c mice (Fig 6B). For two groups of mice, the inoculum was mixed with salivary glands extracts obtained from *Ae*. *albopictus* or *Cx*. *pipiens* mosquitoes. In accordance with what was previously observed after injection of saliva collected from infected mosquitoes, we did not observe any significant difference in the development of JEV pathogenesis in presence of mosquito salivary glands (Fig 6B). Overall these experiments show that European mosquitoes are fully competent at transmitting infectious JEV, but that saliva does not facilitate the development of viral pathogenesis in a susceptible murine model.

## Discussion

In recent years, the increase in locally acquired exotic arbovirus diseases in Europe can be linked to the presence of appropriate combinations of vectors and vertebrate hosts, which could ultimately lead to the establishment of these diseases in Europe [52–54]. Since 2010, sporadic cases of locally acquired chikungunya and dengue fevers have been noted in Europe [55, 56]. The driving forces behind these events are viraemic travelers and the increasing presence of competent vector species, such as *Ae*. *aegypti* and *Ae*. *albopictus*, in temperate regions. Likewise, the circulation of WNV and Usutu virus–two *Flaviviruses*—was reported in 10 European countries [57]. Two studies in Italy reported the infection of local *Cx*. *pipiens* populations with both WNV and Usutu virus [58, 59] and there is an increase in WNV disease incidence in Europe [60]. While JEV RNA was recently detected in mosquitoes and birds in Northern Italy [21, 26], to date, human infections with JEV were only reported in travelers returning from endemic countries [61–63].

Our study shows for the first time that European strains of *Cx*. *pipiens* and *Ae*. *albopictus* are both competent vectors to transmit two genotypes (3 and 5) of JEV. High levels of infection, dissemination and transmission rates were observed in both vectors for either genotypes after oral exposure of mosquitoes to a blood meal containing virus at 8 x 10^6^ FFU/ml. In the present study, we did not evaluate vector competence for viral strains belonging to the genotype 1. Strains belonging to this genotype have displaced the prevalent genotype 3 in several countries in recent years [5–7]. Genotype 1 and 3 strains are genetically close when compared to the more distant genotype 5 strains [8–10]. Since we observed equivalent vector competence of European mosquitoes for genotypes 3 and 5, we hypothesize that those mosquitoes will also be competent at transmitting genotype 1 viruses.

Several mosquitoes from the *Culex* genus are established vectors for JEV. *Cx*. *tritaeniorhynchus* is the main vector in the enzootic cycle of JEV in tropical and subtropical regions of Asia. Interestingly, the vector competence of a *Cx*. *pipiens molestus* population from Taiwan was found to be similar to that of *Cx*. *tritaeniorhynchus*, when tested in laboratory conditions [46], which is in line with our observations. Other investigators reported that *Cx*. *pipiens* populations from other world regions (*Cx*. *pipiens molestus* from Uzbekistan, *Cx*. *pipiens pallens* from Korea, *Cx*. *pipiens* from the United States of America) were less susceptible to JEV and were not always capable of transmitting the virus [50, 64]. Of note, there has been some reports of isolation of JEV from field-caught mosquitoes along the years [12, 17, 21, 65], and all strains of JEV isolated from *Cx*. *pipiens* mosquitoes in Korea in 2012 belonged to the genotype 5 [12]. As strains belonging to the genotype 5 were only rarely isolated, one can wonder if the transmission cycles that are involved in the maintenance of those viruses involve mosquito and amplifying host species different from the established *Cx*. *tritaeniorhynchus /* swine model. Since the currently available vaccines do not confer full protection against JEV genotype 5 strains [66, 67], the risks of JEV g5 transmission to human populations must be carefully examined.

Similarly to *Cx*. *pipiens*, field-collected *Ae*. *albopictus* mosquitoes were occasionally found positive for JEV [22, 68]. Various transmission rates were observed in laboratory settings, from less than 17% for Australian populations [69] to 45% for Taiwanese populations [22]. In our experiments, European *Ae*. *albopictus* was also able to transmit different strains of JEV to high efficiency, which supports the hypothesis that European mosquito populations belonging to these two species have a better vector competence for JEV than populations isolated in other parts of the world.

Our results also showed that the extrinsic incubation period (i.e. the time between ingestion of the virus and the ability of the mosquito to become infectious) for JEV is shorter in *Ae*. *albopictus* than in *Cx*. *pipiens*. We have not performed a formal analysis of the relative life span of our mosquito populations after ingestion of an infectious blood meal. If we assume that both mosquito species have similar life spans, then this would imply that *Ae*. *albopictus* mosquitoes can transmit JEV for a longer period than the French population of *Cx*. *pipiens* and therefore might be a more efficient vector. Specifically, host biting preferences may have consequences on the emergence of the disease in Europe and its transmission dynamics. Arbovirus circulation is defined by many aspects including the population dynamics of the mosquito vector, the extrinsic incubation period, and the population densities of the vertebrate amplifying hosts, all of which are influenced by environmental factors. In the case of JEV, the classic *Cx*. *tritaeniorhynchus*–pig transmission cycle was observed in Japan, a region of high pig farming density, but other species and scenarios could be invoked in regions where pig farming is less abundant, or where *Cx*. *tritaeniorhynchus* is not found [70]. An example of this is the 1995 outbreak of Japanese encephalitis in Australia that involved the presence of domestic pigs and high populations of *Cx*. *annulirostris* [19, 20]. *Ae*. *albopictus* is considered to be an opportunistic feeder: it primarily feeds on mammalian hosts (humans, wild and domestic animals) but can also acquire blood from avian sources [71, 72]. Analysis of feeding patterns in temperate regions showed that populations of *Ae*. *albopictus* in the United States of America mainly fed on mammals and rarely on birds [73]. *Cx*. *pipiens* mosquitoes feed mostly on birds (83%) but also on mammals [74]. Interestingly, it was shown that 20% of *Cx*. *pipiens* emerging from diapause in temperate habitats fed on mammals [73]. Considering the natural cycle of JEV implying birds as reservoir and pigs as amplifying hosts, specificity in host preferences may have consequences on the possible emergence of the disease in Europe and its transmission dynamics. Favorable conditions for JEV emergence may be gathered in several places in Europe where pig breeding sites, bird sanctuaries and *Ae*. *albopictus* and/or *Cx*. *pipiens* mosquitoes coexist (Marquenterre parc, Baie de Somme, France; Camargue, Rhone delta, France; Danube delta, Roumania).

The last part of our study was to investigate the role of mosquito saliva in the transmission of JEV to mice. When insects take a blood meal, they trigger defensive responses from the vertebrate, such as hemostasis and various immune responses. The saliva proteins injected by the mosquito can counteract these defenses, through their angiogenic, anti-hemostatic, anti-inflammatory and immunomodulatory properties [75]. This complex interaction may significantly affect the evolution of the disease; notably co-injection of virus and saliva was shown to potentiate infection of the vertebrate host by arboviruses belonging to various families [49–51, 76, 77]. While we did not observe any enhancement of Japanese encephalitis disease in mice, in presence of salivary gland extract or of saliva collected from either European vectors, further studies are needed to evaluate the impact of saliva on viral burden in different organs. Additionally, we cannot exclude the possibility that JEV pathogenesis might be enhanced by salivary factors of other mosquito species, or that saliva from the two species tested in the present study might enhance pathogenicity in other mammalian species.

## Conclusion

In this study, we have clearly demonstrated that European populations of *Ae*. *albopictus* and *Cx*. *pipiens* were efficient vectors for JEV transmission. Conditions for a putative emergence of JEV in Europe are linked to the possibility for an enzootic cycle to take place in temperate areas. In order to complete the infection cycle, JEV must be transmitted to a susceptible vertebrate host, capable of producing sufficient viral titers for subsequent acquisition by the insect vector. It is therefore important to further investigate whether any European swine or water birds populations can be infected with JEV and produce sufficiently high viremias to infect mosquitoes that feed on them. Such knowledge is critical to assess the potential for JEV to establish local transmission cycles similar to the closely related WNV in Northern Italy.

## Supporting Information

S1 Fig Detection of JEV specific antibodies in inoculated mice. Sera were collected from mice at 28 days post-inoculation, and anti-JEV IgGs were quantified by ELISA using recombinant proteins corresponding to the domain III of JEV g5 envelope protein, as described in [33]. The ELISA absorbance values were measured at 450 nm and the absorbance value obtained from sera of mice inoculated with DPBS is shown as a dashed line (A450 nm = 0.042). Each symbol represents an individual mouse.
(DOCX)
