# Supplementary material for: European Aedes albopictus and Culex pipiens Are Competent Vectors for Japanese Encephalitis Virus
Source: PLoS Negl Trop Dis. 2017 Jan 13;11(1):e0005294. doi: 10.1371/journal.pntd.0005294 (PMC5268654; doi:10.1371/journal.pntd.0005294)
Supplement: S1 Fig — Sera were collected from mice at 28 days post-inoculation, and anti-JEV IgGs were quantified by ELISA using recombinant proteins corresponding to the domain III of JEV g5 envelope protein, as described in [33]. The ELISA absorbance values were measured at 450 nm and the absorbance value obtained from sera of mice inoculated with DPBS is shown as a dashed line (A450 nm = 0.042). Each symbol represents an individual mouse. (DOCX) [file pntd.0005294.s001.docx]

**S1 Figure. Detection of JEV specific antibodies in inoculated mice.**

Sera were collected from mice at 28 days post-inoculation, and anti-JEV IgGs were quantified by ELISA using recombinant proteins corresponding to the domain III of JEV g5 envelope protein, as described in [33]. The ELISA absorbance values were measured at 450 nm and the absorbance value obtained from sera of mice inoculated with DPBS is shown as a dashed line (A_450 nm_ = 0.042). Each symbol represents an individual mouse.
